# A comparative study of well-being, resilience, mindfulness, negative emotions, stress, and burnout among nurses after an online mind–body based intervention during the first COVID-19 pandemic crisis

**DOI:** 10.3389/fpsyg.2023.848637

**Published:** 2023-03-13

**Authors:** Ana Carla Cepeda-Lopez, Leticia Solís Domínguez, Sofía Villarreal Zambrano, Iris Y. Garza-Rodriguez, Alejandra Cortes del Valle, Angélica Quiroga-Garza

**Affiliations:** ^1^Tecnologico de Monterrey, Monterrey, Mexico; ^2^Department of Nutrition, Universidad de Monterrey, San Pedro Garza García, Mexico; ^3^Department of Psychology, Universidad de Monterrey, San Pedro Garza García, Mexico

**Keywords:** well-being, COVID-19, nurses, mindfulness, negative emotion, mind–body

## Abstract

**Background:**

To mitigate against the possible adverse effects of stress among nurses due to the COVID-19 outbreak, we designed a 12-week mind–body based online intervention program to promote well-being and prevent stress-related disorders such as burnout. Our study aimed to compare the impact of the intervention on perception of stress, negative emotions, burnout, mindfulness, resilience, and well-being at pretest and 6 months post-intervention and to compare the effect among nurses working at two different hospitals.

**Methods:**

We conducted an uncontrolled trial using a convenience sample of nurses working at two hospitals in Mexico: one designated to treat confirmed COVID-19 patients (COVID-hospital) and the other whose patients had a negative COVID-19 test on admission (Non COVID-hospital). The 12 week online intervention consisted of 36 mind–body based micropractices, with subjective well-being as the primary outcome. Secondary outcomes were health perception, resilience, mindfulness, negative emotions, stress, and burnout.

**Results:**

A pretest survey was completed by 643 nurses. Of the remaining valid responses, 82% were women, with a mean age of 34.8 (SD = 8.95) years old. For the analysis two groups of nurses were sampled by cluster: a COVID-hospital group of 429 (67%) nurses, and a non-COVID Hospital group of 214 (33%) nurses. The proportion lost to follow-up was 71% at postest (*n* = 188) and 42% at 6 months follow-up (*n* = 371). At pretest, non-COVID hospital nurses had lower subjective well-being and higher burnout than their COVID hospital counterparts. At postest, non-COVID hospital nurses displayed more negative emotions than their COVID hospital peers. At 6 months post-intervention, nurses experienced improved mindfulness, reduced negative emotions and stress, but a decrease in subjective well-being and resilience. Nurses working at the non-COVID hospital had significantly higher mean scores for burnout than those working at the COVID hospital.

**Conclusion:**

The results of our study suggest that our online mind–body interventions can help to reduce stress and negative emotions, yet the effects on subjective well-being and resilience are uncertain. Further research is needed to gain a better understanding of their potential mechanisms and the associated efforts of such online interventions.

**Clinical Trial Registration:**

ClinicalTrials.gov, NCT05515172.

## 1. Introduction

Historically nurses have played a critical role in addressing the medical and psychological needs of patients during outbreaks, including coronavirus disease 2019 (COVID-19), severe acute respiratory syndrome (Shih et al., 2009), the Middle East respiratory coronavirus (Choi and Kim, 2016), zika (Wilson and Nguyen, 2017; Dran, 2018), and Ebola virus disease (Kollie et al., 2017; Raven et al., 2018). Before the COVID-19 pandemic, the World Health Organization (WHO) designated 2020 as the International Year of the Nurse and the Midwife (World Health Organization, World Health Day, 2020). The aim was to celebrate these essential healthcare professionals and bring to light the challenges often inherent in the profession to ensure they get the support they need. Nurses are the healthcare team members who spend the most time with patients and report the highest levels of job stress (World Health Organization, 2020). Research in nursing practice during the past decade has shown that long work hours, dealing with pain, loss, and emotional suffering, caring for dying patients and providing support to families is not new nor only due to the COVID-19 pandemic (Lazarus and Folkman, 1984; Andolhe et al., 2015)_._ Facing this critical situation, made nurses more susceptible to distress and burnout (Raudenská et al., 2020).

As the impact of the COVID-19 outbreak began to manifest on patients and healthcare systems, nurses across the world were asked to do the unthinkable, pushing body and mind to the limit (Maben and Bridges, 2020)_._ During this time nurses worldwide were required to work recurrent long hours and draining shifts, sometimes with limited access to personal protective equipment and evolving guidance on how to care for patients infected with the virus. Moreover, nurses faced separation from their families, stigma, fear of being infected, fear of infecting their loved ones, and pain of losing patients and colleagues (El-Hage et al., 2020; Walton et al., 2020). These extreme conditions triggered depression, anxiety, insomnia, phobia, obsessive–compulsive symptoms, and somatization symptoms in nurses worldwide (Cai et al., 2020).

Given the adverse environmental demands related to the COVID-19 outbreak there were significant concerns about nurses’ mental health and psychological adaptation worldwide (Sampaio et al., 2021). A study performed in Mexico showed that many nurses were suffering from psychological disturbances due to the COVID-19 outbreak. The authors showed that 47% of nurses reported moderate–severe traumatic distress response, 42% of nurses reported a high level of emotional exhaustion, and 42% showed moderate–severe psychological distress (Cortés-Álvarez et al., 2020). A stressful situation is one in which the demands of the setting threaten to exceed the resources of the individual (Lazarus and Folkman, 1984)_._ Although stress responses evolved as adaptive processes, Selye observed that severe, prolonged stress responses might lead to tissue damage and disease (Selye, 1956). Environmental demands can overwhelm an individual’s resources, and therefore when high levels of stress are perceived, this can damage health leading to burnout (Montero-Marín et al., 2014). Burnout syndrome is a common psychological phenomenon among nurses characterized by the inability to cope with chronic occupational stress adapt to or protect oneself from it (Maslach et al., 2001; Johnson et al., 2018). This can be the result of too much effort in the workplace with limited opportunities for recovery.

Fundamentally, resilience refers to the ability to maintain or regain mental health successfully despite experiencing adversity (Jackson et al., 2007). Definitions have evolved as scientific knowledge has increased. New perspectives share “a focus on conceptualizing resilience at multiple levels, from the biological to the social and policy level, a focus on the dynamic nature of resilience itself as a fluid, interacting process of adaptation, and a move away from conceptualizing resilience as an individual trait” (Denckla et al., 2020). In line with this conceptualization, existential positive psychology is based on positive change brought upon by personal strength, higher appreciation of life, maintaining hope, existential courage, engagement in meaningful activities, a relationship with others, and the possibility of new opportunities in life (Wong, 2010). It also considers the negative side of life as a counterbalance to the positive (Wong, 2011; Wong, 2020). It establishes that suffering brought on by trauma, death, and disease is inevitable. Nonetheless, adversity functions as promoter of personal and spiritual development achieving post-traumatic growth. This is based on positive change brought upon by personal strength, higher appreciation of life, relationship with others, and the possibility of new opportunities in life (Magne et al., 2021).

Using mind–body interventions has been shown to be key to promoting resilience to chronic stress. Mind–body interventions cover a range of practices that trigger the “relaxation response,” a physiological state of lower sympathetic tone, increased parasympathetic tone, and lower resting heart rate, respiratory rate, and blood pressure (Benson et al., 1974; Cozzolino et al., 2020). For example, the practice of meditation has been shown to physically change regions of the brain, improving information-processing and emotion-regulation abilities (Grossman et al., 2004; Davidson and Lutz, 2008)_._ Research on mindfulness, a psychological construct drawn from the Buddhist tradition, has identified that mindfulness-based programs designed to train individuals to cultivate mindfulness and incorporate its practice into daily life decreases rumination (Kabat-Zinn, 2003). According to Bishop et al. (2004), mindfulness encompasses two components: self-regulation of attention, and adoption of a particular orientation toward one’s experiences. Current conceptualizations point to two primary, essential elements of mindfulness: awareness and nonjudgmental acceptance of one’s moment-to-moment experience. A possible explanation for the roll of mindfulness practices in reducing reactivity to emotional stimuli and enhancing psychological well-being is that it promotes disengagement from perseverative cognitive activities and enhances attentional capacities through gains in working memory. These cognitive gains, in turn, contribute to effective emotion-regulation strategies and the development of skills to deal with negative thoughts and emotions in an adaptive and flexible manner (Bishop et al., 2004). Cognitive reappraisal (i.e., cognitive–behavioral approaches) involves reframing an emotional stimulus to change its emotional impact (Gross, 2015). It has been shown that for individuals who are struggling with acute elevations in psychological distress (the COVID-19 outbreak), reappraisal may be an important first line of defense, whereas acceptance may be beneficial in the longer term, perhaps after more immediate emotional relief has occurred (Troy et al., 2018).

A combination of physical and psychological characteristics, including body chemistry and personality factors, gives individuals the skills to be stress-resilient. Indeed, previous research has demonstrated the positive effects of resilience programs in nurses (Foster et al., 2018; Henshall et al., 2020). Resilience-based interventions, focused on healthcare workers, have emerged during the COVID-19 pandemic using online tools such as video calls and applications. Findings from this study demonstrated that resilience enhancement programs can increase nurses’ levels of resilience and confidence and improve inter-professional relationships (Weiner et al., 2020). Moreover, relaxation exercises such as yoga and qigong practices have been found to be effective in reducing chronic pain or anxiety associated with stressful experiences (Griffith et al., 2008; Zhang et al., 2021). Thus, interventions that combine physical and psychological mind–body practices could be effective in promoting physical and emotional well-being across nurses.

As part of efforts to mitigate against the adverse effects of stress among nurses due to the COVID-19 outbreak, we designed an online mind–body based intervention combining physical and psychological mind–body practices such as mindfulness to mitigate psychological distress associated with the pandemic. Therefore, the current investigation examined whether the 12 weeks mind–body based online intervention could decrease the perception of stress and improve the perception of subjective well-being in nurses working in two different setting during the first wave of the COVID-19 pandemic.

## 2. Materials and methods

### 2.1. Study design, setting, and participants

**Figure 1:**
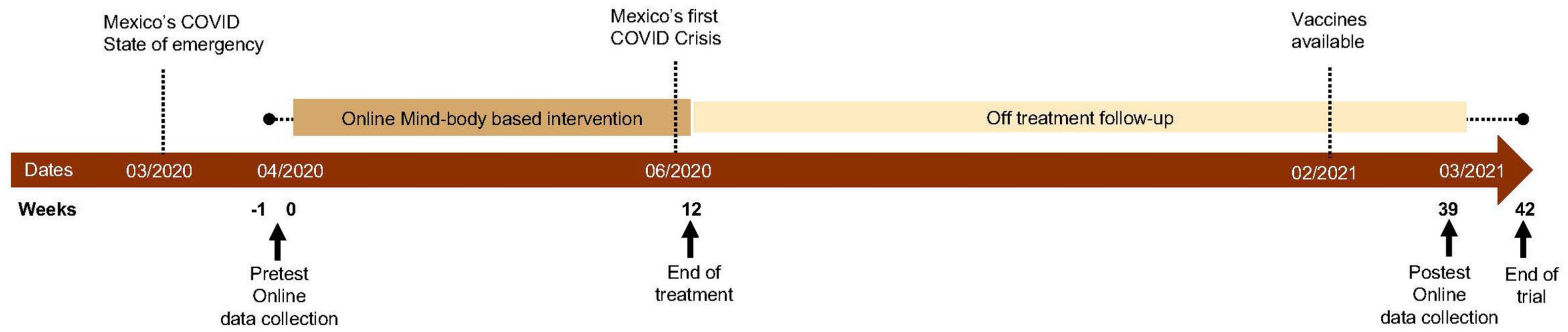
Participant Timeline.

This uncontrolled trial used a convenience sample at two private hospitals in northeast Mexico: a COVID-19 designated hospital that treated confirmed COVID-19 patients (COVID-hospital) and a hospital were patients had to demonstrate a negative COVID-19 test on admission (Non COVID-hospital). Mexico declared a state of emergency due to the COVID-19 pandemic on March 30th, 2020. Our intervention was implemented from April 22nd, 2020, until July 12th, 2020 (12 weeks). During this first wave of the pandemic, we invited nurses to answer a survey and to participate in a 12 weeks-long mind–body based intervention. Figure 1 describes the overlap of the study’s intervention implementation and data acquisition time points with the COVID-19 pandemic. The inclusion criteria for the participants were as follows: (a) nurses who were currently employed in one of the selected private hospitals and (b) nurses who provided informed consent. Participation was voluntary and participants did not receive any compensation.

Study participants were informed about the intervention *via* word of mouth. The questionnaire links were sent in an institutional email. All participants who agreed to participate in the study signed a consent form before answering the online survey with demographic questions and self-report measures to assess perception of health, subjective well-being, resilience, mindfulness, negative emotions, stress, and burnout. The completion of the online survey took approximately 25 min.

A total of 769 nurses working at two hospitals during the onset of COVID-19 in Mexico were invited to participate in an intervention and to complete a survey at three-time points. A pretest was conducted during the onset of COVID-19 in Mexico, with a response rate of 84%. Subsequently, postest measures were conducted during the peak of the first COVID-19 crisis in Mexico (de México, 2020), with a response rate of 29%. Follow-up measures were conducted 6 months later, when the first COVID-19 vaccines had been made available to health workers in the area and the lowest number of COVID-19 cases in the last year was reported (Gobierno de Nuevo Leon, 2021), with a response rate of 58%. Our analysis comparing the efficacy of the intervention was limited to participants who completed the pre-test and follow-up measures. The study variables questionnaire was designed as an online survey using Google Forms (https://www.google.com/intl/en-GB/forms/about/; Alphabet Co., Mountain View, CA, United States). The Institutional Ethical Committee of the School of Medicine of the Universidad de Monterrey approved the study (CEI-002-20191210).

### 2.2. Description of the online intervention

A multidisciplinary team of experts, experienced in the practice and teaching of mind–body techniques, developed a 12-week online intervention to address COVID-19-related chronic stress and promote well-being among nurses. This intervention was composed of a toolbox of well-being resources, containing 36 micro-practices. Frontline nurses were incorporated into the research team that developed the study intervention, which was an essential aspect of the project. Informal interviews were conducted with nurses to identify the most effective methods to adapt the intervention to the context. Nurse leaders provided local support by sharing micro-practices three times per week in a group setting, with the practices being done collectively. Since not all micro-practices were going to resonate with every nurse, the goal was that the nursing staff could also access the link with the toolbox of well-being resources using their personal devices (laptops/tablets/smartphones) so they could select those that they wished to engage with at any moment.

**Figure 2:**
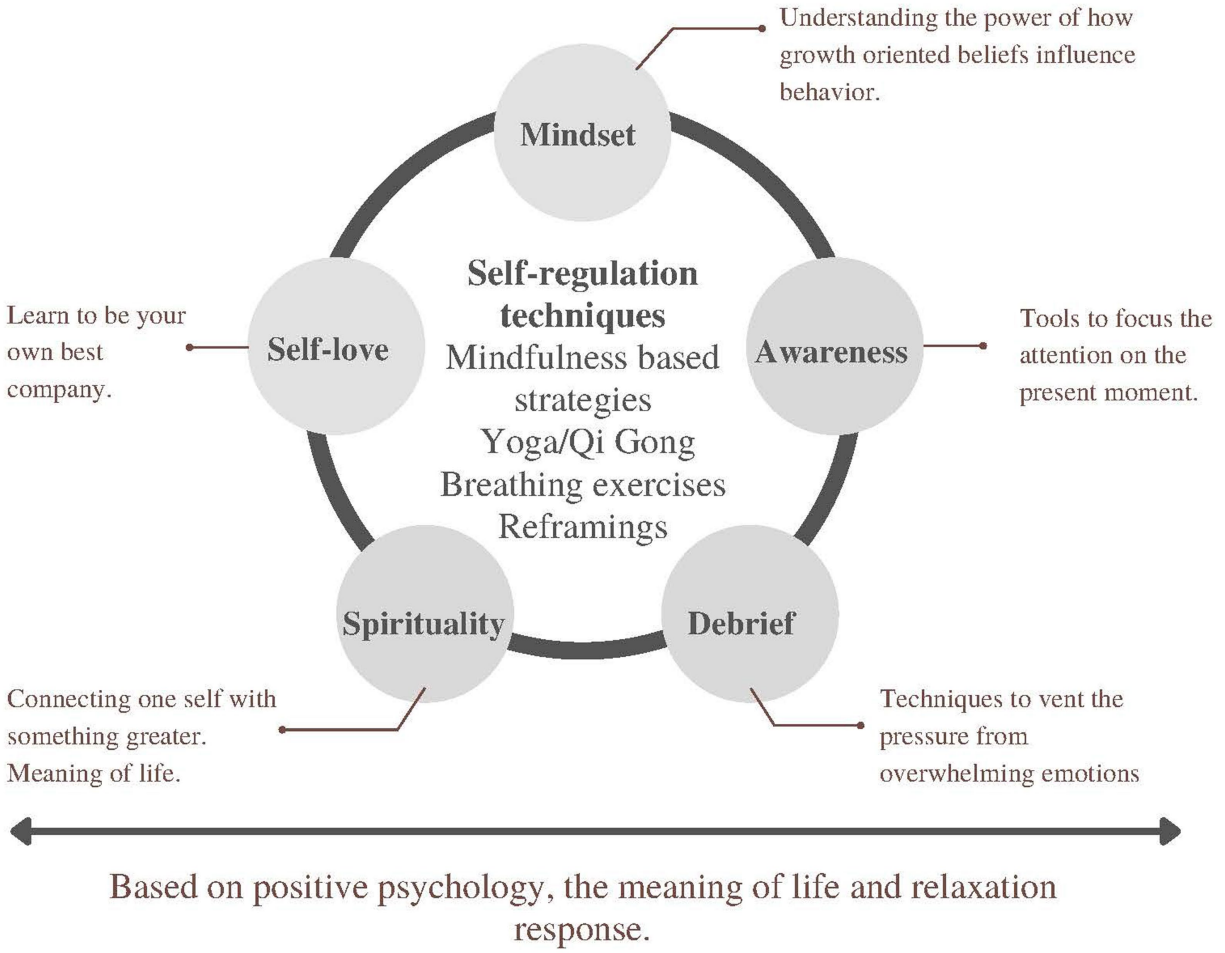
Description of the intervention.

The micro-practices were delivered for 12 weeks using a web-based platform administered to nursing staff by the Nurse Manager using a WhatsApp group. Each link presented the following structure: (a) reflection on the micro-practice carried out during the previous week, (b) presentation and explanation of the role of the micro-practice on well-being, (c) a 3-min YouTube video or audio file that participants could watch or listen to, including different techniques to elicit the relaxation response such as mindfulness-based stress reduction, single-focus meditation, self-regulation exercises (i.e., yoga qi-gong), breathing practices (i.e., diaphragmatic breathing), awareness practices (attentiveness to perceptual impressions in one’s environment, as well as internal cues, such as bodily sensations, thoughts, and emotions), spirituality and reframing strategies based of existential positive psychology (realizing connections with the self, finding meaning/purpose through acceptance, courage, letting it go, maintaining calm with whatever comes, and connecting with something greater than oneself), and journaling (Figure 2). Micro-practices were designed to be quick (only required a few seconds to a few minutes to implement) and could be done solo or with others, and almost anywhere. Most of the micro-practices were linked to an existing activity, such as practicing mindfulness when using hand sanitizer. Participants that were particularly at risk for experiencing significant mental distress were identified. They were assigned to a weekly cohesion group directed by a health coach using the Zoom platform. The cohesion group served as a compassionate accompaniment to mitigate acute anguish *via* Psychological First Aid to increase social engagement and peer support. Additionally, nurses were offered further evaluation with psychological support at the hospital.

### 2.3. Quantitative research methods

#### 2.3.1. Demographic data

We assessed demographic data such as age, gender, marital status, hospital, department, education, and children at home/parental status.

#### 2.3.2. Subjective well-being

We used the Spanish translation of the PERMA Profiler developed by Butler and Kern (2016) and translated by Tarragona. The PERMA Profiler measures five domains of flourishing: positive emotion, engagement, relationships, meaning, and accomplishment. Each domain has three items, with the total score from these domains constituting a measure of subjective well-being. Subjective well-being provides a helpful index on how we are doing and how well we live individually. PERMA Profiler also includes additional items that evaluate negative emotion (three items), perceived physical health (three items), and loneliness (one item, excluded from the study). The measure consists of 23 items. Questions are on an 11-point response scale ranging from 0 to 10, with the end points labeled. (Cronbach’s Alpha: Subjective well-being α = 0.886; Health α = 0.856; Negative Emotions α = 0.814).

#### 2.3.3. Resilience

We used the Spanish version (Rodríguez-Rey et al., 2016) of the Brief Resilience Scale (BRS; Smith et al., 2008) aiming to assess individuals’ ability to recover from stressful circumstances. The scale consists of six Likert scale items that range from strongly disagree (Shih et al., 2009) to strongly agree (Raven et al., 2018; Cronbach’s α = 0.612).

#### 2.3.4. Mindfulness, attention, and awareness

We employed the Spanish version (Barajas and Garra, 2014) of the Mindfulness Attention Awareness Scale (MAAS; Brown and Ryan, 2003) which measures characteristics of mindfulness, namely, open, or receptive awareness and attention to the present. The MAAS consists of 15 items where participants establish the frequency, they find themselves in that situation. The answers are recorded using a six-point scale that ranges from almost always (Shih et al., 2009) to almost never (Raven et al., 2018). Higher scores indicate greater mindfulness (Cronbach’s α = 0.912).

#### 2.3.5. Perceived stress

We used the Spanish version (Brito Ortíz et al., 2020) of the Perceived Stress Scale (PSS; Cohen et al., 1983) which measures the degree to which unpredictable or unexpected situations and events in the last month were considered stressful for the participants. The PSS is a 14-item questionnaire with a five-point response scale ranging from 0 (never) to 4 (very often). A higher score indicates higher stress (Cronbach’s α = 0.819).

#### 2.3.6. Burnout

The Burnout Clinical Subtypes Questionnaire (BCSQ-12; Montero-Marín et al., 2011) consists of 12 items equally distributed between the dimensions manifested through the differentiation of three clinical subtypes: (Shih et al., 2009) Frenetic, which is characterized by “overload” and the perception of jeopardizing one’s health to pursue worthwhile results, and is highly associated with exhaustion; (Choi and Kim, 2016) under-challenged, which is characterized by “lack of development,” defined as the perception of a lack of personal growth, together with the desire for a more rewarding occupation that better corresponds to one’s abilities, and is most strongly associated with cynicism; and (Dran, 2018) worn-out, which is characterized by “neglect,” defined as an inattentive and careless response to responsibilities, and is closely associated with inefficacy. The BCSQ-12 provides an approach to burnout, focusing on identifying the type of dissatisfaction and discomfort experienced. Participants had to state their degree of agreement with each affirmation using a seven-point Likert scale from totally disagree (Shih et al., 2009) to totally agree (World Health Organization, World Health Day, 2020; Cronbach’s Alpha: Burnout α = 0.857; Overload α = 0.803; Lack of Development α = 0.801; Negligence α = 0.768).

### 2.4. Statistical analysis

Computed statistical analysis and visualizations were performed using the statistical packages SPSS (Statistical Package for the Social Sciences; IBM 2017; Version 28). Internal consistency statistics were analyzed for all instruments. Data were checked for a normal distribution. For data that complied with a normal distribution, the mean and standard deviation (*SD*) are reported. Chi-squared test were used to assess differences in categorical demographic variables between hospitals. Independent samples *t* tests were used to compare COVID and non-COVID hospitals on all outcome variables at pretest, posttest, and 6-month follow-up. Due to the low response rate at posttest, when testing the intervention we compared pretest and follow-up scores only. Paired-samples *t* tests were used to assess changes in outcome variables from pretest to follow-up among the 371 participants who completed both surveys. Cohen’s *d* is reported as an effect size measure. This analysis was repeated for the COVID and non-COVID hospital subsamples. If the data were skewed, the non-parametric Wilcoxon signed-ranks test was used. The alpha level was set to 0.05 for all analyses.

## 3. Results

From the 769 nurses invited to participate, 643 (84%) completed the pretest survey (83% women, *M_age_* = 34.05, *SD* = 9.22). For the analysis two groups of nurses were sampled by cluster: COVID-hospital group consisted of 429 (67%) nurses, and non-COVID Hospital group consisted of 214 (33%) nurses. The demographic characteristics are shown in Table 1. Age, having children at home, education, and department were significantly different between groups. At postest measurements, 188 valid responses were obtained (COVID-hospital nurses *n* = 144; non-COVID Hospital nurses *n* = 44). The proportion lost to follow-up was 71%. At 6 months post-intervention measurements, 371 valid responses were obtained (COVID-hospital nurses *n* = 255; non-COVID Hospital nurses *n* = 116). The proportion lost to follow-up was 42%.

**Table 1:** Baseline characteristics for the whole sample and stratified by hospital.

Variable		Whole sample (*n* = 643)	COVID hospital (*n* = 429)	Non-COVID Hospital (*n* = 214)	*p* value
Age (y) Mean (SD)		34.1 (9.2)	35.0 (9.6)	32.1 (8.0)	< 0.001
Gender %	Male	17.4	15.9	20.6	0.138
	Female	82.6	84.1	79.4	
Marital Status %	Single	50.4	49.7	51.9	0.687
	Married	43.9	43.8	43.9	
	Divorced	5.1	5.8	3.7	
	Other	0.6	0.7	0.5	
Children at home % Education %	Yes	53.4	54.5	50.9	0.033
	No	46.6	45.5	49.1	
	Bachelor	52.1%	50.6%	55.1%	0.015
	Specialty	20.2%	17.5%	25.7%	
	Technical degree	27.7%	31.9%	19.2%	
	Other	4.8%	5.8%	2.8%	
Department %	Surgery	19.3%	20.3%	17.3%	< 0.001
	Internal medicine	15.4%	13.8%	18.7%	
	Intensive care unit	9.5%	8.9%	10.7%	
	Emergency	9.0%	7.0%	13.1%	
	Other	46.9%	50%	40.2%	

COVID-hospital: nurses working at a COVID-19 designated hospital that treated confirmed COVID-19 patients.

Non COVID Hospital: nurses working at a hospital were patients had COVID-19 negative test on admission.

Between hospitals comparison: continuous data = *t*-test; categorical data = chi-square.

y = years: SD = standard deviation.

Mean scores of perceived health, subjective well-being, resilience, mindfulness, stress, burnout, and negative emotions were assessed at different time points for the entire sample of nurses, and the results were compared between subgroups of COVID and Non-COVID Hospital nurses (Table 2). At pretest, non-COVID hospital nurses had significantly lower mean scores for subjective well-being and higher burnout scores compared to COVID hospital nurses. At postest, the mean score for negative emotions was significantly higher for non-COVID hospital nurses than for COVID hospital nurses. At 6 months follow-up, non-COVID hospital nurses had significantly higher mean scores for burnout than COVID hospital nurses.

**Table 2:** Mean scores of perceived health, subjective well-being, resilience, mindfulness, stress, burnout, and negative emotions for the whole sample and divided by COVID Hospital and Non-COVID Hospital nurses at pretest, postest, and 6-month follow-up.

Variables		Pretest mean (SD)	*p*	*d*	Postest mean (SD)	*p*	*d*	Follow-up mean (SD)	*p*	*d*
Perceived Health	Whole sample	23.20 (4.64)			22.24 (6.24)			23.09 (4.97)		
	COVID Hospital	23.41 (4.61)	0.092	0.141	22.88 (5.90)	0.010	0.447	23.35 (4.62)	0.132	0.169
	Non-COVID Hospital	22.76 (4.68)			20.14 (6.90)			22.51 (5.65)		
Subjective Well-being	Whole sample	134.37 (15.67)			117.93 (23.90)			126.95 (15.38)		
	COVID Hospital	135.42 (15.04)	0.016	0.202	119.06 (22.26)	0.239	0.203	126.31 (15.35)	0.235	−0.133
	Non-COVID Hospital	132.27 (16.71)			114.20 (28.58)			128.36 (15.42)		
Resilience	Whole sample	22.23 (3.03)			22.38 (2.90)			21.10 (3.87)		
	COVID Hospital	22.34 (3.00)	0.202	0.107	22.44 (2.77)	0.610	0.088	20.97 (3.88)	0.319	−0.112
	Non-COVID Hospital	22.02 (3.10)			22.20 (3.29)			21.40 (3.85)		
Mindfulness	Whole sample	63.28 (9.64)			63.08 (9.67)			64.00 (10.03)		
	COVID Hospital	63.80 (9.65)	0.051	0164	63.67 (9.43)	0.128	0.263	64.22 (10.24)	0.535	0.070
	Non-COVID Hospital	62.22 (9.58)			61.14 (10.29)			63.52 (9.60)		
Stress	Whole sample	22.28 (6.58)			22.02 (6.79)			20.22 (7.22)		
	COVID Hospital	22.08 (6.69)	0.275	−0.091	21.78 (6.97)	0.400	−0.145	20.11 (7.14)	0.668	−0.048
	Non-COVID Hospital	22.68 (6.34)			22.77 (6.18)			20.46 (7.40)		
Burnout	Whole sample	36.26 (10.66)			40.56 (12.07)			35.34 (12.00)		
	COVID Hospital	35.39 (10.30)	0.003	−0.248	40.94 (12.10)	0.437	0.134	34.34 (10.63)	0.016	−0.272
	Non-COVID Hospital	38.02 (11.17)			39.32 (12.00)			37.55 (14.09)		
Negative Emotions	Whole sample	14.21 (6.47)			12.06 (7.08)			13.01 (6.81)		
	COVID Hospital	13.88 (6.30)	0.070	−0.152	11.44 (7.03)	0.029	−0.379	12.77 (6.83)	0.324	−0.111
	Non-COVID Hospital	14.86 (6.78)			14.09 (6.94)			13.53 (6.77)		

*p* value indicates whether the observed differences between the hospitals at the same time point are statistically significant. *d* = Cohen’s d.

*p*, p value. Pretest: Whole sample (*n* = 643); COVID Hospital (*n* = 429); Non-COVID Hospital (*n* = 214). Postest: Whole sample (*n* = 188); COVID Hospital (*n* = 144), and Non-COVID Hospital (*n* = 44). Follow up: Whole sample (*n* = 371); COVID Hospital (*n* = 255) and Non-COVID Hospital (*n* = 116).

In a subsample of 371 nurses, who completed pre-test and 6 months follow-up surveys, the mean scores of subjective well-being, resilience, stress, and negative emotions significantly decreased from pre-test to 6 months follow-up, while the mean score for mindfulness increased. The mean score for the perception of health and burnout, however, remained stable. A different trend was observed among non-COVID Hospital nurses, where only the mean scores of subjective well-being and stress decreased, while the mean scores of mindfulness, resilience, and negative emotions remained unchanged (Table 3).

**Table 3:** Mean scores and mean difference for perceived health, subjective well-being, resilience, mindfulness, stress, burnout and negative emotions at pretest and 6 months follow-up for a sub-sample of nurses who completed both timepoint measures and divided by hospitals.

Variables		Pretest mean (SD)	Follow-up mean (SD)	Mean difference (SD)	*p*	*d*
Perceived Health	Sub-sample	23.07 (4.65)	23.09 (4.97)	−0.01 (4.50)	0.954	0.00
	COVID hospital	23.13 (4.54)	23.35 (4.62)	−0.22 (4.17)	0.402	−0.05
	Non-COVID hospital	22.95 (4.88)	22.51 (5.65)	0.44 (5.15)	0.360	0.09
Subjective Well-being	Sub-sample	134.09 (15.83)	126.95 (15.38)	7.14 (14.98)	<0.001	0.48
	COVID hospital	134.61 (14.78)	126.31 (15.35)	8.30 (14.29)	<0.001	0.58
	Non-COVID hospital	132.95 (17.94)	128.36 (15.42)	4.59 (16.18)	0.003	0.28
Resilience	Sub-sample	22.14 (2.87)	21.10 (3.87)	1.04 (4.08)	<0.001	0.25
	COVID hospital	22.15 (2.81)	20.97 (3.88)	1.19 (3.86)	<0.001	0.31
	Non-COVID hospital	22.10 (3.01)	21.40 (3.85)	0.71 (4.52)	0.095	0.16
Mindfulness	Sub-sample	62.67 (9.85)	64.00 (10.03)	−1.33 (9.62)	0.008	−0.14
	COVID hospital	63.05 (10.04)	64.22 (10.24)	−1.17 (8.95)	0.038	−0.13
	Non-COVID hospital	61.84 (9.42)	63.52 (9.57)	−1.67 (10.98)	0.104	−0.15
Stress	Sub-sample	22.55 (6.34)	20.22 (7.22)	2.33 (7.01)	<0.001	0.33
	COVID hospital	22.74 (6.33)	20.11 (7.14)	2.63 (6.87)	<0.001	0.38
	Non-COVID hospital	22.13 (6.37)	20.46 (7.40)	1.67 (7.30)	0.015	0.23
Burnout	Sub-sample	36.15 (10.39)	35.34 (11.90)	0.81 (11.58)	0.178	0.07
	COVID hospital	35.51 (10.14)	34.34 (10.63)	1.17 (10.20)	0.067	0.12
	Non-COVID hospital	37.57 (10.84)	37.55 (14.09)	0.02 (14.18)	0.990	0.00
Negative Emotions	Sub-sample	14.45 (6.40)	13.01 (6.81)	1.44 (6.28)	<0.001	0.23
	COVID hospital	14.53 (6.15)	12.77 (6.83)	1.76 (6.17)	<0.001	0.29
	Non-COVID hospital	14.27 (6.95)	13.53 (6.77)	0.74 (6.47)	0.219	0.12

Differences in the means of the scores of the variables pretest-versus 6 months follow-up were analyzed using a paired-samples *t*-test. *p*, *p* value; *d*, Cohen’s *d*.

Pretest and follow up: Sub sample (*n* = 371); COVID Hospital (*n* = 255), and Non-COVID Hospital (*n* = 116).

## 4. Discussion

The results of our study indicate that an online mind–body based intervention was associated with improved mindfulness and decreased negative emotions and stress among nurses during the first wave of the COVID-19 pandemic. However, this intervention was also associated with a decrease in subjective well-being and resilience. There were no significant changes in burnout and health perception. At 6 months post-intervention, nurses working at the non-COVID hospital had significantly higher mean scores for burnout than those working at the COVID hospital.

After 6 months post-intervention, nurses in both COVID and non-COVID hospitals showed an increase in mindfulness and a decrease in perceived stress and negative emotions. Notably, nurses in the COVID Hospital who had higher levels of mindfulness also presented lower scores in negative emotions compared to those in the non-COVID hospital. Under the Perceived Stress Scale (PSS; Cohen et al., 1983) we measured situations appraised as stressful and recorded as unpredictable, uncontrollable, and overloaded in nurses’ lives; that is, when the individual’s resources to cope with stressors are disabled and a sense of imbalance in control over health, relations, or emotions negatively impacting well-being (Schönfeld et al., 2016; Wersebe et al., 2018; Kun, 2022). Mindfulness practices involve directing one’s awareness to the present moment without judgment, in order to lessen stress and negative emotions when faced with stressful situations (Kabat-Zinn, 2005; Antonova et al., 2021). In this way, it can enable nurses to interpret circumstances as less distressing, which in turn can decrease negative emotions. It is possible that our findings were due to nurses paying more mindful attention to their appraisals of challenging situations, which enabled them to adapt better to stress, resulting in a decrease in negative emotions and an increase in mindfulness (Weinstein et al., 2009). Our results are compatible with well-documented effects on the effectiveness of mindfulness-based interventions for nurses resulting in the reduction of stress (Guillaumie et al., 2017). The beneficial effects of mindfulness on the reduction of stress perception have primarily been reported for face-to-face programs. Recent studies showed that online mindfulness interventions can successfully reduce stress and anxiety while improving emotion regulation in participants (Sanilevici et al., 2021; Ju et al., 2022). Our results could also be explained by the temporal nature of the data collection in the context of the COVID-19 pandemic. Pretest measures were collected 1 month after the official outbreak of the pandemic, which may have caused alarm and elevated levels of stress and negative emotions in nurses. Follow-up data was collected after vaccines became available for health staff Mexico, which may have resulted in a reduction of perceived stress and negative emotions due to a decreased sense of risk. Our findings support the feasibility online mind–body based intervention for the promotion of mindfulness, reduction of stress and negative emotions, especially during difficult times like the COVID-19 pandemic. However, further research is needed to investigate this relationship in a context where the circumstances are not rapidly changing, and with the inclusion of a control group.

Based on our observation of an increase in mindfulness and a decrease in perceived stress and negative emotions, we anticipated that the perception of resilience and well-being would also increase over time. However, this was not the outcome. Nurses decrease in resilience scores could be more directly linked to their incapacity to recover from the health-related outcomes of the COVID-19 crisis. This could be attributed to the fact that the Brief Resilience Scale (Smith et al., 2008) measures an individual’s ability to recover from stress rather than resilience as a personality trait, which is reflected in scales such as the Connor-Davidson Resilience Scale (Connor and Davidson, 2003). Moreover, nurses’ perception of subjective well-being, as measured under the PERMA approach, considers that higher levels of the five components (positive emotions, engagement, relationships, meaning, and achievement) act as a buffer against negative emotions and distress (Seligman, 2018). It is proposed that, despite advances in well-being research, there is a lack of a unifying framework that clarifies dimensions of well-being that can be improved through training. Additionally, research is needed to understand the mechanisms through which these interventions work and to identify any potential hindering intervening factors associated with their use. In order to ensure that these interventions can be used safely and effectively, it is important to conduct further research to answer these questions.

Overall, we found that perception of burnout remained stable over time. We observed that nurses not directly involved in the care of COVID-19 patients had higher scores of negative emotions and burnout compared to those working in COVID hospitals, both at the initial assessment and during the subsequent follow-up assessment, which differed from the findings of previous research (Hu et al., 2020; Sarboozi Hoseinabadi et al., 2020). A possible explanation for this unexpected trend is that nurses at the COVID-hospital were likely more adequately equipped to fulfill their job requirements in comparison to those employed in the non-COVID hospital. Moreover, non-COVID hospitals nurses had increased workload, reassignment to other roles or duties. In a recent study conducted in China during the COVID-19 pandemic, they found that frontline medical staff had a lower frequency of burnout as compared with those working in usual wards. Frontline staff are the individuals on the frontlines of the pandemic, providing direct care to patients with COVID-19, while those in usual wards are providing care to patients with other medical conditions who are not affected by the virus. In that study frontline workers were less worried about becoming infected despite working directly with infected patients (Wu et al., 2020). Another possible explanation is that by directly addressing COVID-19 patients, nurses may have felt a greater sense of control of their situation as well as increasing recognition. Control in the workplace is thought to be a major driver of engagement and important for avoiding burnout (Maslach and Leiter, 2016). Our results confirm that in the face of a crisis, both frontline and usual health staff should be considered when policies and procedures to support the well-being of health care workers are planned.

There are several limitations of this study. An important limitation of our study is the lack of a control group. As the pandemic caused fluctuating conditions for each time period, it cannot be determined whether the differences in nurses’ responses were due to the intervention or the impact of the COVID-19 crisis. Nurses worked at a private institution, limiting the generalization of our findings to nurses working at public healthcare settings in Mexico that may have had different experiences of the COVID-19 outbreak. Given the high rate of attrition, our results should be viewed with caution. Over 70% of nurses did not fill out the post-test, and 42% did not take the 6-month post-intervention survey. This could lead to response bias, as those who did not respond may have been too stressed or we may have only received responses from highly engaged nurses. Finally, we were unable to objectively measure the adherence to the intervention. To gain insight into the implementation of the program, weekly check-ins with nurses’ managers were conducted. Furthermore, we monitored the user count and the view count for the web-based platform where the intervention was conducted. But unfortunately, we were not able to accurately measure the degree to which individual participants engaged with the program or how frequently they practiced.

Despite the limitations noted above, the results of this study indicate that our online mind–body intervention has the potential to enhance mindfulness and lessen stress and negative emotions. However, its influence on the subjective well-being and resilience of hospital staff nurses is uncertain. This paper contributes to the existing literature by providing insight into nurses’ experiences with combined mind–body based interventions, which has been under-researched. Our results may enable future comparative work to further understand what works for these professionals and may enhance evidence-based development of new initiatives to support their well-being. Additionally, our findings suggest that both frontline and usual health care workers should be considered when developing strategies to maintain the health and safety of health care personnel during times of crisis.

## Data availability statement

The raw data supporting the conclusions of this article will be made available by the authors, without undue reservation.

## Ethics statement

The studies involving human participants were reviewed and approved by The Institutional Ethical Committee of the School of Medicine of the Universidad de Monterrey approved the study (CEI-002-20191210). The patients/participants provided their written informed consent to participate in this study.

## Author contributions

AC-L, AQ-G and AV generated the idea. AC-L designed the intervention. AC-L and SV were responsible for drafting the manuscript. AC-L, SV, AV, IG-R, and LS coordinate the study and participants. AC-L, SV, AV, IG-R, LS, and AQ-G critically revised it for important intellectual content, gave final approval to the finished manuscript, and agreed to be accountable for all aspects of the work. All authors contributed to the article and approved the submitted version.

## Conflict of interest

The authors declare that the research was conducted in the absence of any commercial or financial relationships that could be construed as a potential conflict of interest.

## Publisher’s note

All claims expressed in this article are solely those of the authors and do not necessarily represent those of their affiliated organizations, or those of the publisher, the editors and the reviewers. Any product that may be evaluated in this article, or claim that may be made by its manufacturer, is not guaranteed or endorsed by the publisher.
